# Endoscopic band ligation is safe despite low platelet count and high INR

**DOI:** 10.1007/s12072-023-10515-y

**Published:** 2023-04-06

**Authors:** Nikolaus Pfisterer, Michael Schwarz, Mathias Jachs, Florian Putre, Lukas Ritt, Mattias Mandorfer, Christian Madl, Michael Trauner, Thomas Reiberger

**Affiliations:** 1 Division of Gastroenterology and Hepatology, Department of Internal Medicine III, Medical University of Vienna, Vienna, Austria; 2 Vienna Hepatic Hemodynamic Lab, Medical University of Vienna, Vienna, Austria; 3 4. Medizinische Abteilung für Gastroenterologie und Hepatologie, Klinik Landstrasse, Vienna, Austria; 4 4. Medizinische Abteilung für Gastroenterologie und Hepatologie, Klinik Ottakring, Vienna, Austria; 5 Private Medical School, Sigmund Freud University, Vienna, Austria; 6 Christian-Doppler Laboratory for Portal Hypertension and Liver Fibrosis, Medical University of Vienna, Vienna, Austria

**Keywords:** Endoscopic band ligation, Procedure related bleeding, Variceal bleeding, Portal hypertension, Liver cirrhosis

## Abstract

**Background:**

Prophylactic endoscopic band ligation (EBL) is used to prevent variceal bleeding in patients with liver cirrhosis. The association of thrombocytopenia, high INR (international normalized ratio) and liver dysfunction with the risk of procedure-related bleeding (PRB) remains debated and recommendations are controversial.

**Methods:**

We analyzed real-life data of cirrhotic patients undergoing elective EBL at two large Viennese centers between Q1/2000–Q1/2018. PRB was defined as bleeding occurring within 30 days after EBL.

**Results:**

We included 617 patients undergoing a total of 1178 prophylactic EBL procedures (median 2 per patient). Sixteen (2.6%) of 617 patients experienced PRB after a median of 12.5 (IQR 17.3) days with no difference in characteristics and laboratory values between the two groups. The proportion of patients with platelets (PLT) < 50 G/L or INR ≥ 1.5 was similar in patients with vs. without PRB. A higher MELD showed a non-significant association with EBL-related bleeding risk (odds ratio, OR 1.07; 95% confidence interval 95% CI 1.00–1.16, p = 0.058). While serum bilirubin was a significant predictor for PRB (OR: 1.10; 95% CI 1.03–1.18), the presence of large varices (OR 0.85 vs. small varices; 95% CI 0.20–3.84), INR (OR 0.50; 95% CI 0.10–3.14), PLT (OR 1.00; 95% CI 1.00–1.01) and the use of non-selective betablockers (OR 1.20; CI 95% 0.38–3.76) were not associated with PRB.

**Conclusion:**

EBL is safe and procedure-related bleedings are rare (2.6%) including in patients with thrombocytopenia < 50 G/L or high INR ≥ 1.5. Only high MELD, and especially high bilirubin seem to be linked to an increased risk of EBL-related bleeding.

## Introduction

Esophageal variceal (EV) bleeding is a severe complication of portal hypertension in patients with liver cirrhosis and the bleeding-related mortality is still high up to 20% [1–5]. To avoid (re-)bleeding episodes in primary and secondary prophylaxis, current guidelines recommend treatment with non-selective betablockers (NSBBs) or/and endoscopic band ligation (EBL) [6–10]. This approach has been shown to effectively reduce bleeding and mortality rates [11–16].

Furthermore, EBL is also a standard treatment for acute variceal bleeding, but if it fails, self‐expandable metal stent and/or rescue transjugular intrahepatic portosystemic shunt (TIPS) are indicated [7, 9, 17].

EBL is generally safe and has a low rate of procedural risk in the treatment of EV and is safer compared to other endoscopic therapies, such as injection sclerotherapy [18, 19].

Esophageal ulcers may form after EBL, but are usually only superficial and heal within 2–3 weeks [20].

Compared to NSBB, EBL is associated with a lower rate of adverse events, however, potential adverse events of EBL could be more severe and life-threatening (e.g. EBL-related ulcer bleeding) [21, 22]. Bleeding from banding ulcers represents  the main severe complication of EBL and has been described to occur in 2.3% to 10% of patients [22–31]. The role of potential risk factors (e.g. liver function, platelets count, number of rubber bands placed, etc.) for PRB has not yet been entirely clarified, but a better understanding of these factors could help to avoid such complications. Furthermore, no consensus has been established  on the actual risk factors for PRB and their management  [7, 9, 32].

The aim of this retrospective multicenter study was to assess potential risk factors for procedure-related bleeding (PRB) within 30 days after first elective/prophylactic EBL in patients with liver cirrhosis and portal hypertension.

## Patients and methods

### Study design

This retrospective study was conducted in two tertiary clinical centers (Vienna General Hospital of the Medical University of Vienna and Klinik Landstrasse in Vienna) including patients with liver cirrhosis and portal hypertension. Patients undergoing endoscopic band ligation (EBL) for esophageal varices (EV) were included between January 2000 and May 2020. Inclusion criteria were the presence of EV at endoscopy, elective EBL, age > 18 years and diagnosis of liver cirrhosis.

Patients with non-cirrhotic portal hypertension receiving EBL, other than EBL endoscopic treatment, previous transjugular intrahepatic portosystemic shunt (TIPS) implantation or orthotopic liver transplantation, occlusive portal vein thrombosis, patients with fundal varices only, patients with acute and/or uncontrolled variceal bleeding at baseline and insufficient medical/endoscopic records were excluded from this study. However, patients with hepatocellular carcinoma at baseline who fulfilled the Milan criteria were included in this study [33].

We intentionally excluded patients undergoing emergency EBL (i.e. therapeutic EBL) performed to treat active gastrointestinal bleeding, since this setting would have not allowed us to discriminate EBL-related (i.e. strictly procedure-related) bleeding from early rebleeding.

The baseline characteristics were compared between a group with a perceived lower bleeding risk (platelets > 50 and international normalized ratio (INR) < 1.5, respectively) and a group with a perceived increased bleeding risk (platelets ≤ 50 G/L or INR ≥ 1.5, respectively, Tables 1 and 2). Patients with no available platelets and/or INR values at baseline were excluded from this study.

**Table 1:** Patient characteristics stratified according to platelet count

	Platelets ≤ 50 G/L	Platelets > 50 G/L	p value
Patients (n,%)	92 (14.9)	525 (85.1)	
Age (years, median, IQR)	53.1 (15.4)	58.7 (15.9)	0.001
Sex (m/f, %m)	71/21 (77.2)	349/176 (66.5)	0.052
Aetiology of cirrhosis			
Alcohol (n, %)	35 (38)	280 (53.3)	0.005
Viral hepatitis (n, %)	31 (33.7)	92 (17.5)	
Alcohol + viral hepatitis (n, %)	3 (3.3)	26 (5)	
Other (n, %)	8 (8.7)	52 (9.9)	
Cryptogenic (n, %)	15 (16.3)	75 (14.3)	
Large varices ≥ 5 mm (n, %)	80 (87)	451 (85.9)	0.872
Procedure-related bleeding (n,%)	1 (1.3)	13 (2.9)	0.276
Small varices < 5 mm (n, %)	8 (8.7)	57 (10.9)	0.712
Procedure-related bleeding (n,%)	1 (12.5)	1 (1.6)	0.705
Additional gastric varices (n, %)	3 (3.3)	9 (1.7)	0.401
Serum creatinine (mg/dL, IQR)	0.8 (0.3)	0.9 (0.3)	0.128
Serum albumin (g/dL, IQR)	34.9 (8.6)	33.8 (8.9)	0.311
Serum bilirubin (mg/dL, IQR)	2 (2.1)	1.6 (1.9)	0.007
MELD (IQR)	13 (8)	12 (6)	0.015
Ascites			
None (n, %)	45 (48.9)	211 (40.2)	0.115
Moderate (n, %)	37 (40.2)	204 (45.7)	
Severe (n, %)	10 (10.9)	100 (19)	
Child–Pugh stage			
CPS A (n, %)	36 (39.1)	165 (31.4)	0.059
CPS B (n, %)	23 (25)	189 (36)	
CPS C (n, %)	19 (20.7)	78 (14.9)	
NSBB use (n, %)	66 (71.7)	372 (70.9)	0.902
AST (U/L, IQR)	42.5 (44.3)	46 (46.5)	0.845
ALT (U/L, IQR)	29.5 (25.5)	29 (26)	0.652
GGT (U/L, IQR)	65 (94)	100 (136)	0.001
Number of endoscopies/year	2.8 (8.6)	2.8 (7)	0.643
Number of EBL session/year	1.6 (7.4)	1.3 (7)	0.715

EBL (endoscopic banding ligation), NSBB (non-selective betablockers), IQR (interquartile range), m (male), f (female), mm (millimeters), n (total numbers), mg/dL (milligram per deciliter), INR (International Normalized Ratio), MELD (Model for End-Stage Liver Disease), AST (Aspartate transaminase), ALT (Alanine transaminase), GGT (Gamma-glutamyl transferase), U/L (unit per liter), FU (Follow Up)

**Table 2:** Patient characteristics stratified according to INR

	INR < 1.5	INR ≥ 1.5	p value
Patients (n,%)	458 (74.2)	159 (25.8)	
Age (years, median, IQR)	59 (16.6)	53.6 (15.6)	0.001
Sex (m/f, %m)	310/148 (67.7)	110/49 (69.2)	0.768
Aetiology of cirrhosis			
Alcohol (n, %)	214 (46.7)	101 (63.5)	0.001
Viral hepatitis (n, %)	99 (21.6)	24 (15.1)	
Alcohol + viral hepatitis (n, %)	19 (4.1)	10 (6.4)	
Other (n, %)	48 (10.6)	10 (7.5)	
Cryptogenic (n, %)	78 (17)	12 (7.5)	
Large varices ≥ 5 mm (n, %)	393 (85.8)	138 (86.8)	0.894
Procedure-related bleeding s (n, %)	11 (2.8)	3 (2.2)	0.999
Small varices < 5 mm (n, %)	48 (10.5)	17 (10.7)	0.999
Procedure-related bleeding (n, %)	1 (2.1)	1 (5.9)	0.458
Additional gastric varices (n, %)	9 (2)	3 (1.9)	0.999
Serum creatinine (mg/dL, IQR)	0.9 (0.3)	0.8 (0.4)	0.007
Serum albumin (mg/dL, IQR)	34.8 (8.4)	31.1 (8.8)	0.001
Serum bilirubin (mg/dL, IQR)	1.4 (1.5)	2.8 (3.7)	0.001
MELD (IQR)	11 (4)	17 (7)	0.001
Ascites			
None (n, %)	212 (46.3)	44 (27.7)	0.001
Moderate (n, %)	169 (36.9)	72 (45.3)	
Severe (n, %)	72 (15.7)	38 (23.9)	
Child–Pugh stage			
CPS A (n, %)	177 (38.6)	24 (15.1)	0.001
CPS B (n, %)	168 (36.7)	43 (27)	
CPS C (n, %)	37 (8.1)	60 (37.7)	
NSBB use (n, %)	326 (71.2)	112 (70.4)	0.919
AST (U/L, IQR)	43 (41)	55.5 (50.8)	0.002
ALT (U/L, IQR)	29.5 (27)	29 (25)	0.765
GGT (U/L, IQR)	103 (143.5)	82 (107.5)	0.086
Number of endoscopies/year	2.8 (6.8)	3.1 (8.7)	0.652
Number of EBL session/year	1 (0.3)	1.3 (5.8)	0.780

EBL (endoscopic banding ligation), NSBB (non-selective betablockers), IQR (interquartile range), m (male), f (female), mm (millimeters), n (total numbers), mg/dL (milligram per deciliter), INR (International Normalized Ratio), MELD (Model for End-Stage Liver Disease), CPS (Child–Pugh score), AST (Aspartate transaminase), ALT (Alanine transaminase), GGT (Gamma-glutamyl transferase), U/L (unit per liter)

Our aim was to determine factors associated with procedural-related bleeding (PRB) as defined as any clinically significant episode of hematemesis, melena or both occurring within 30 days after elective EBL.

### Parameters

In this study laboratory (aspartate transaminase, alanine transaminase, gamma-glutamyl transferase, serum-bilirubin, prothrombin time, international normalized ratio and platelet count), endoscopic (size of varices, presence of additional gastric varices, presence of red spots and bleeding during examination), clinical parameters (age, sex, etiology of cirrhosis, presence and grade of ascites, and presence and grade of hepatic encephalopathy) were extracted from medical records. During further follow-up, we recorded specific clinical outcomes, i.e. acute variceal bleeding, TIPS implantation, liver transplantation and death (including its cause). Early variceal (re-)bleeding after EBL was defined as the presence of haematemesis and/or clinical and laboratory evidence of acute blood loss from esophageal varices, which occurred within 30 days from the first EBL. Important to note, that other (portal-hypertensive) bleeding events occurring later during follow-up were not counted as PRB.

### Statistics

Laboratory, endoscopic, clinical parameters and specific data on clinical outcomes were collected. Continuous variables were reported as median with interquartile range (IQR) and categorical variables were reported as absolute numbers (and proportions, %) of patients.

Comparisons of continuous variables in Tables 1 and 2 (age, creatinine, serum albumin, serum bilirubin, liver enzymes, MELD, number of endoscopies and EBL per year) were performed using Student t test or Mann–Whitney U-test, as applicable. Chi-square or Fisher’s exact test was used for group comparisons of categorical variables (sex, etiology of cirrhosis, size of varices, Child–Pugh score/grade, grade of ascites, number of additional gastric varices and use of NSBB) in Tables 1 and 2.

Kaplan–Meier curves were used to visualize procedure-related bleeding (PRB) rates within 30 days according to potential risk factors, such as the size of varices, serum bilirubin, INR and platelet count—and group comparisons were performed using the log-rank test.

Potential liver-related risk factors (serum bilirubin, creatinine, albumin, platelet count, Child–Pugh score, MELD, size of varices, alcohol intake, use of non-selective betablockers and the number of rubber bands placed in the respective EBL procedures), as well as age and sex, were included into a Cox regression model to assess their effect on EBL-related bleeding. The odds ratio (OR) including the 95% confidence interval (95%CI) was calculated for each individual risk factor. In order to identify independent predictors for PRB multivariate Cox regression models were performed. A p value ≤ 0.05 was considered statistically significant. IBM SPSS statistics Version 28 (SPSS Inc., Armonk, New York, USA) and GRAPHPAD Prism 9 (GRAPHPAD Software, La Jolla, California, USA) were used for statistical analyses.

## Results

### *Patient characteristics *(Fig. 1, Tables 1, 2)

**Fig. 1:**
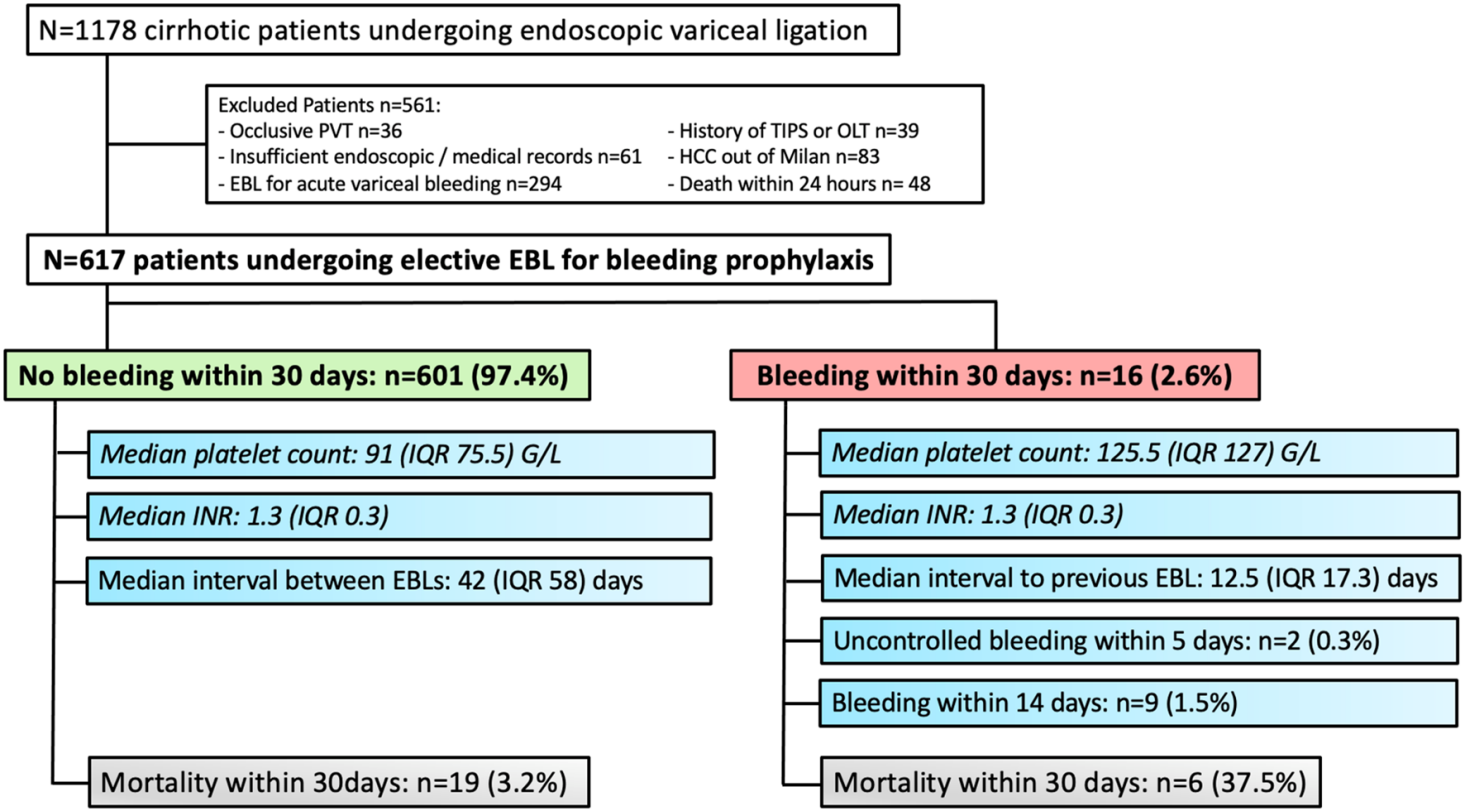
Patient flow chart: Among 1178 patients undergoing endoscopic band ligation (EBL), 617 patients with only elective EBL were finally included in this study. 16 patients (2.6%) developed bleeding within a median of 12.5 days after EBL. Six patients (37.5%) died within 30 days because of procedure-related bleeding. n (total numbers), PH (portal hypertension), IQR (interquartile range), PVT (portal vein thrombosis), HCC (hepatocellular carcinoma), OLT (orthotopic liver transplantation), TIPS (transjugular intrahepatic portosystemic shunt), EBL (endoscopic band ligation), INR (international normalized ratio)

A total of 1178 patients underwent endoscopic treatment for esophageal varices (EV). After the exclusion of 561 patients, a total number of 617 patients were included in this study.

Among the 16 (2.6%) patients, who experienced procedure-related bleeding (PRB) within 30 days after the first elective endoscopic band ligation (EBL), 9 (1.5%) patients bled within 14 days and bleeding-related mortality was observed in 6 patients (37.5%), 2 of them (0.3%) owing to uncontrolled bleeding. The mortality within 30 days in patients who did not bleed within 30 days was lower with n = 19 (3.2%). A higher median platelet count of 125.5 G/L (interquartile range, IQR 127) was observed compared to patients, who did not bleed within 30 days (91 G/L, IQR 75.5) and the median INR was the same in both groups (Bleeding within 30 days: 1.3 IQR 0.3 vs. no bleeding within 30 days: 1.3 IQR 0.3). Interestingly, the median interval to the previous EBL after PRB was 12.5 (IQR 17.3) days and the median interval between the EBLs in patients with no PRB was 42 (IQR 58) days.

The median overall follow-up of our patient cohort was 28 (IQR 42.8) months; importantly, PRB was only evaluated in the first 30 days after elective EBL. PRB in the first 30 days occurred in patients with large and in patients with small varices were 3.1% (2/65) and 2.6% (14/531). The rate of PRB was not significantly different (p = 0.691). In patients in which PRB occurred, the number of previous EBL sessions was significantly lower than in patients without PRB (1 IQR 1 vs. 2 IQR 1, p = 0.001). However, the number of rubber bands being placed within 30 days was comparable in both groups (PRB: 5 IQR 2 vs. No PRB: 5 IQR 2, p = 0.532).

The baseline characteristics were stratified into 2 groups: one group with a perceived lower bleeding risk with platelets > 50 or INR < 1.5, respectively and one group with a perceived increased bleeding risk with platelets ≤ 50 G/L or INR ≥ 1.5, respectively. A total number of n = 92 had thrombocytopenia with platelets ≤ 50 G/L and n = 525 patients had platelet counts > 50 G/L. Alcohol intake was the most common cause of liver cirrhosis. Patients with platelets ≤ 50 G/L had significantly higher MELD (13 IQR 8 vs. 12 IQR 6, p = 0.015), especially higher serum bilirubin levels (2.0 IQR 2.1 vs. 1.6 IQR 1.9 mg/dL, p = 0.007). Otherwise, patients with platelets > 50 G/L were older (53.1 IQR 15.4 vs. 58.7 IQR 15.9 years, p = 0.001) and had higher GGT values (65 IQR 94 vs. 100 IQR 136 IU/L, p = 0.001). Importantly, there were no significant differences between patients with platelets ≤ 50 G/L vs. platelets > 50 G/L regarding other laboratory parameters, sex, size of varices, presence of additional gastric varices, Child–Pugh score, presence of ascites, use of NSBB, and the number of endoscopies or EBL sessions per year. The rate of PRB in patients with large varices (1.3% vs. 2.9%, p = 0.276) and patients with small varices (12.5% vs. 1.6%, p = 0.705) were not statistically different between patients with platelets ≤ 50 G/L and platelets > 50.

When we stratified patients according to INR and compared baseline characteristics, alcohol intake was the most common cause of liver cirrhosis in both groups. Patients with INR < 1.5 had significantly higher serum creatinine values (0.9 IQR 0.3 vs. 0.8 IQR 0.4 mg/dL, p = 0.007) and were older than patients with INR ≥ 1.5 (59.0 IQR 16.6 vs. 53.6 IQR 15.6 years, p = 0.001). On the other side, patients with INR ≥ 1.5 had a significantly higher MELD (11 IQR 4 vs.17 IQR 7, p = 0.001). Lower serum albumin (34.8 IQR 8.4 vs. 31.1 IQR 8.8 g/dL, p = 0.001) and higher bilirubin (1.4 IQR 1.5 vs. 2.8 IQR 3.7 mg/dL, p = 0.001) were seen in patients with INR ≥ 1.5. There were no significant differences in other characteristics between patients with INR < 1.5 and INR ≥ 1.5, except for AST (43 IQR 41 vs. 55.5 IQR 50.8, p = 0.002). Furthermore, the PRB were also not significantly different regarding high/low INR when assessing patients with large varices (2.8% vs. 2.2%, p = 0.999) or small varices (2.1% vs. 5.9%, p = 0.470) separately.

### Risk factors associated with procedure-related bleeding within 30 days after elective endoscopic band ligation (Table 3, Fig. 2)

**Table 3:** Risk factors for EBL procedure-related bleeding

	No bleeding within 30 days	EBL-related bleeding	Univariate odds ratio (95% CI)	p value	Multivariate odds ratio (95% CI)	p value
Patients (n, %)	601 (79.4)	16 (2.6)				
Age (median, IQR)	57.9 (16.3)	57.8 (18.3)	1.00 (0.96–1.04)	0.807		
Sex (m/f, %m)	408/193 (67.9)	12/4 (75)	1.42 (0.45–4.46)	0.548		
ALD (n,%) etiology	307 (51.1)	8 (50)	0.96 (0.36–2.59)	0.932		
NSBB (n, %)	426 (70.9)	12 (75)	1.20 (0.38–3.76)	0.758		
Large Varices > 5 mm (n,%)	531 (88.4)	14 (87.5)	0.85 (0.20–3.84)	0.836		
Number of rubber bands placed (IQR)	5 (2)	5 (2)	1.08 (0.79–1.48)	0.634		
Platelets (10^9^/L, IQR)	91 (75.5)	125.5 (127)	1.00 (1.00–1.01)	0.296		
Platelets ≤ 50 G/L (n, %)	88 (14.6)	2 (12.5)	0.81 (0.18–3.63)	0.784		
INR (IQR)	1.3 (0.3)	1.3 (0.3)	1.01 (0.21–4.91)	0.992	0.50 (0.10–3.14)	0.502
INR ≥ 1.5 (n, %)	155 (25.8)	4 (25)	1.10 (0.31–3.02)	0.943		
Bilirubin (mg/dL, IQR)	1.6 (1.9)	2.4 (5.8)	1.10 (1.02–1.17)	0.009	1.10 (1.03–1.18)	0.007
Creatinine (mg/dL, IQR)	0.8 (0.3)	0.8 (0.4)	1.16 (0.75–1.80)	0.500	1.15 (0.72–1.84)	0.563
Albumin	33.9 (8.9)	33.9 (10.9)	1.03 (0.95–1.11)	0.491		
MELD	12 (6)	14 (11)	1.07 (1.00–1.16)	0.058		
Child Score	7 (3)	8 (4)	1.12 (0.89–1.41)	0.330		
Child-Stage C (n, %)	93 (15.5)	4 (25)	1.56 (0.49–5.02)	0.452		

This table shows a comparison of characteristics of patients without bleeding within 30 days versus patients experiencing bleeding within 30 days after EBL. Percentage (%) refers to the total (n) of patients in the respective subgroup; creatinine (Serum creatinine), continuous variables are expressed as median with interquartile range (IQR). The odds ratio for bleeding risk was calculated by univariate followed by a multivariate binary logistic regression model

NSBBs (non-selective betablockers), m (male), f (female), %m (percentage of men), ALD (alcohol-related liver disease), INR (international normalized ratio), 95% CI (95% confidence interval), MELD (Model for End-Stage Liver Disease)

**Fig. 2:**
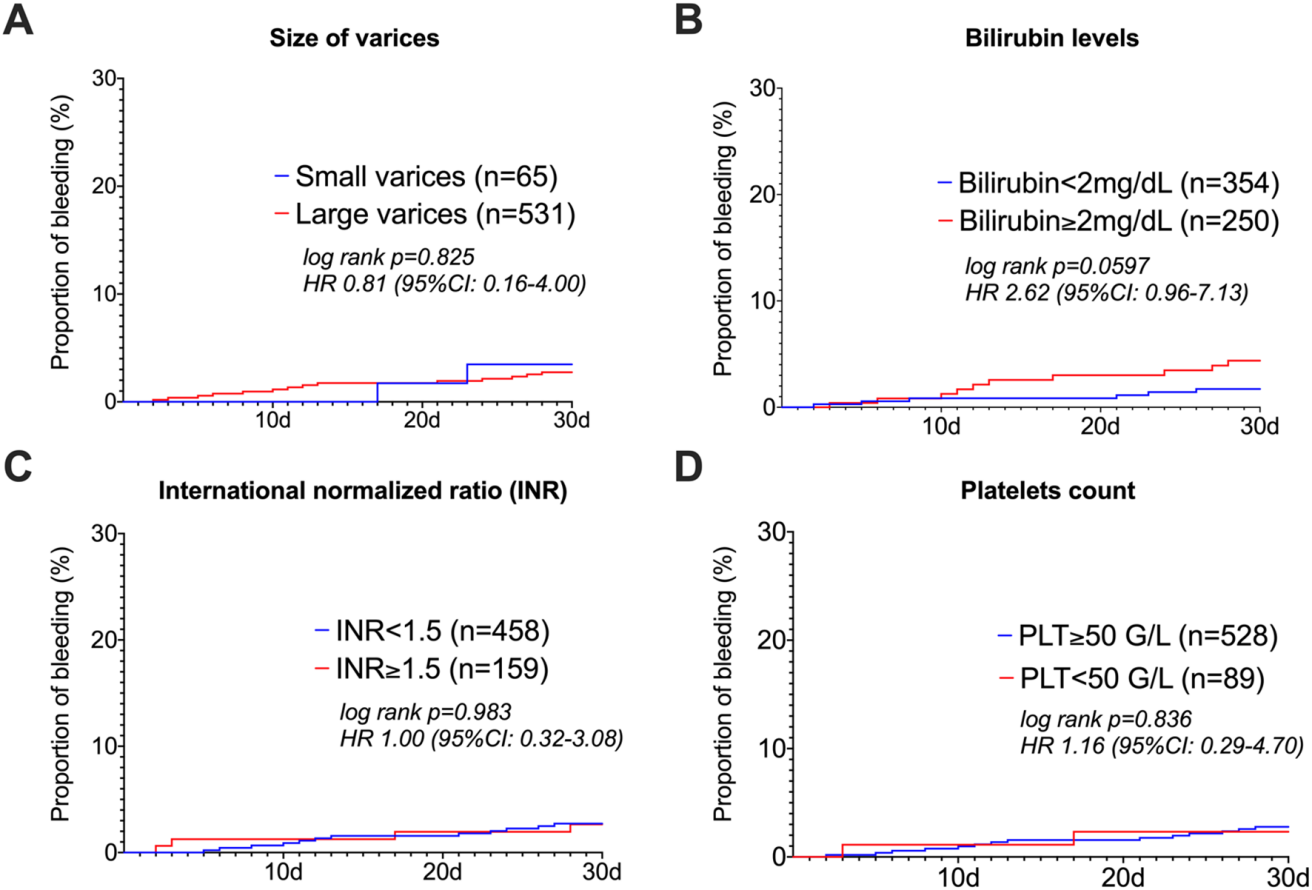
EBL-related bleeding rates. Kaplan–Meier curves of procedure-related bleeding events according to A size of varices, B serum bilirubin, C international normalized ratio, and D platelet count within 30 days after EBL. n (total numbers), d (days), PLT (platelets count in 10^9^/L), INR (international normalized ratio), mg/dL (milligram per deciliter), HR (Hazard-Ratio; statistical comparisons were performed by log-rank tests)

Higher MELD showed a trend to a higher risk of PRB (odds ratio, OR 1.07, confidence interval 95% CI 1.00–1.16, p = 0.058; Table 3). Neither the Child–Pugh score (OR 1.12, 95% CI 0.89–1.41, p = 0.330) nor Child-stage C (OR 1.56, 95% CI 0.49–5.02, p = 0.452) were significantly associated with a risk for PRB.

Serum bilirubin levels were associated with a higher risk of PRB within 30 days on univariate analyses (OR 1.10, 95% CI 1.02–1.17, p=0.009) and on multivariate analysis (OR 1.10, 95% CI 1.03–1.18, p = 0.007). The Kaplan Meier analysis showed a trend towards higher bleeding rates of patients with a high bilirubin level of ≥ 2 mg/dL (hazard ratio HR 2.62, 95% CI 0.96–7.13, log-rank p = 0.0597, Fig. 2B).

Other MELD components, i.e. serum creatinine (OR 1.16, 95% CI 0.75–1.80, p = 0.500) and INR (OR 1.01, 95% CI 0.21–4.91, p=0.992) were no significant risk factors for bleeding. Serum albumin was not a significant risk factor for PRB (OR 1.03, 95% CI 0.95–1.11, p = 0.491).

Patients with an INR ≥ 1.5, did not show higher bleeding rates in our Kaplan Meier analyses (HR 1.00, 95% CI 0.32–3.08, log-rank p = 0.983, Fig. 2C) and did not show a higher risk of PRB in the univariate analyses (OR 1.10, 95% CI 0.31–3.02, p = 0.943).

We found no association between bleeding and large varices undergoing banding (OR 0.85 95% CI 0.20–3.84, p = 0.836). Similarly, a higher post-EBL bleeding risk in patients undergoing banding of large varices was not confirmed by Kaplan Meier (HR 0.81, 95% CI 0.16–4.00, log-rank  p = 0.825, Fig. 2A). Furthermore, the number of rubber bands being placed was not a risk factor for PRB (OR 1.08, 95% CI 0.79–1.48, p=0.634).

Concerning platelet count, no significant link between PRB within 30 days and the platelet count (OR 1.00, 95% CI 1.00–1.01, p = 0.296) was found. Thrombocytopenia of < 50 G/L was also neither associated with a higher rate of PRB on Kaplan-Maier analysis (HR 1.16, 95% CI 0.29–4.70, log-rank p = 0.836, Fig. 2D) nor a risk factor for PRB (OR 0.81, 95% CI 0.18–3.63, p=0.784).

Furthermore, the use of NSBB (OR 1.20, 95% CI 0.38–3.76, p = 0.758), alcohol-related cirrhosis (OR 0.96, 95% CI 0.36–2.59, p=0.932), male sex (OR 1.42, 95% CI 0.45–4.46, p = 0.548) or age (OR 1.00, 95% CI 0.96–1.04, p=807) were all not associated with PRB events within 30 days after EBL.

## Discussion

We assessed procedure-related (re-)bleeding (PRB) rates within 30 days after elective endoscopic band ligation (EBL) in a large real-life cohort of patients with liver cirrhosis treated at two major liver units. The result of this study showed that only bilirubin was a strong predictor for PRB and remained an independent risk factor for bleeding within 30 days after EBL after adjustment for covariates. Other MELD components, which are surrogate parameters for the severity of liver disease, such as creatinine and INR were not associated with a higher risk of (re-)bleeding within 30 days.

Comparable to our study, Drolz et al. [23] reported risk factors for early (presumably procedure-related) variceal bleeding after elective EBL in 444 patients with liver cirrhosis. In their study [23], bleeding was observed in 38 (5%) patients within 30 days and elevated bilirubin was associated with a 50% increased risk of (hazard ratio 1.5), which is in line with our study. High bilirubin was also reported as a risk factor for EBL-related bleeding in other studies [23, 34] Along the same line, impaired hepatic function—as reflected by higher MELD, low prothrombin time and Child–Pugh stage C—has been linked to PRB risk in several previous reports [21, 27, 29, 30, 33].

The size of varices correlates with the portal pressure; therefore, a larger size of varices may be predictive of (re-)bleeding risk [35–37]. Our real-life data showed that the size of varices was not associated with PRB. Furthermore, in our study the rate of PRB in patients with large vs. small varices, was not significantly different (p = 0.691). In contrast, some other studies [23, 38] found a larger variceal size (grade III/IV according to Paquet) as a risk factor for (re-)bleeding within 30 days of prophylactic EBL.

Furthermore, a recent mucosal injury due to a banding ulcer may be a relevant risk factor for PRB (especially when more rubber bands are placed in the same session), however, the impact of the time interval between two prophylactic EBL procedures on 30-day bleeding remains largely unknown. Interestingly, although there were no significant differences between patients with PRB and without PRB within 30 days regarding the number of rubber bands being placed (p = 0.532), patients who did suffer from PRB had significantly lower previous EBL sessions than patients without bleeding within 30 days (p = 0.001).

One study [28] demonstrated a high aspartate transaminase-to-platelet ratio index (APRI) to be an independent risk factor for PRB. Interestingly, our study found no significant link between PRB within 30 days and the platelet count. Furthermore, a lower platelet count < 50 G/L was also not associated with a higher rate of PRB. These results show that EBL is safe for patients with low platelet counts < 50 G/L. These results were also in line with several other studies [22, 23, 30, 31].

Non-selective betablockers (NSBB) are recommended to be used in all patients with clinically significant portal hypertension (CSPH) in order to prevent first hepatic decompensation (i.e. acute variceal bleeding, development of ascites…) and mortality [6, 7, 9, 12, 39, 40].

The rate of using NSBB for prophylaxis in this study around 70% seems low. Still, most patients in primary prophylaxis (130/434, 30%) were treated only with endoscopic band ligation (EBL) which is aligned with current guidelines [7, 9]. However, as we report on real-life, retrospective data, we can only speculate on the reasons. Importantly, we have previously conducted a survey among Austrian physicians and found that 47.1% of the surveyed persons would use NSBB for primary prophylaxis and 87.1% would perform combined treatment with NSBB and EBL for secondary prophylaxis of variceal bleeding [41]. Thus, it seems that despite the recommendation to preferentially use NSBB for primary prophylaxis and the combination of NSBB and EBL in secondary prophylaxis, many physicians (or endoscopists) do not use NSBB according to their own preference. In our specific study setting, the use of NSBB had no significant effect on PRB. Interestingly, another prospective study including 175 patients showed a lower risk of PRB with NSBBs [31].

In our cohort, 16 (2.6%) of 617 patients experienced PRB and 6 patients (37.5%) died in the further course after elective EBL. These results confirm the low bleeding rate and good safety of EBL in previous studies [22, 23, 28–31]. Interestingly, the median time from previous EBL to PRB was 12.5 days and more than half of the patients bled within 14 days. Thus, PRB mainly occurs after a short time following the elective procedure and the patients should be informed because they are usually discharged from the hospital one day after the procedure.

The retrospective design of this study represents an important limitation. However, the results are based on a large real-life patient sample. Adherence to specifically dietary recommendations after EBL was not recorded, which could have affected the rebleeding rates [42–44].

In conclusion, elective EBL is a very safe procedure with a low risk of procedure-associated bleedings even in patients with low platelet counts < 50 G/L or with INR > 1.5.
